# Supplementary material for: Crawling and Gliding: A Computational Model for Shape-Driven Cell Migration
Source: PLoS Comput Biol. 2015 Oct 21;11(10):e1004280. doi: 10.1371/journal.pcbi.1004280 (PMC4619082; doi:10.1371/journal.pcbi.1004280)
Supplement: S1 Code — (ZIP) [file pcbi.1004280.s012.zip › release/tst/doc/html/classCell.html]

Tissue Simulation Toolkit: Cell Class Reference


Tissue Simulation Toolkit
 0.1.4.1


- Main Page
- Namespaces
- Classes
- Files

- Class List
- Class Hierarchy
- Class Members

Public Member Functions |
Static Public Member Functions |
Public Attributes |
Protected Attributes |
Static Protected Attributes |
Friends |
List of all members

Cell Class Reference

`#include <cell.h>`

Collaboration diagram for Cell:

[legend]

|  |  |
| --- | --- |
| Public Member Functions | |
|  | Cell (const Dish &who, int settau=1) |
|  | Constructor to insert a cell into Dish "who". More... |
|  | |
|  | Cell (void) |
|  | |
|  | ~Cell (void) |
|  | |
|  | Cell (const Cell &src) |
|  | Default copy constructor. More... |
|  | |
| void | CellBirth (Cell &mother) |
|  | Add a new cell to the dish. More... |
|  | |
| Cell & | operator= (const Cell &src) |
|  | Assignment operator. More... |
|  | |
| bool | AliveP (void) const |
|  | Returns false if Cell has apoptosed (vanished). More... |
|  | |
| int | Colour (void) const |
|  | Returns the cell colour. More... |
|  | |
| void | setTau (int settau) |
|  | Set cell type of this Cell. More... |
|  | |
| int | getTau (void) |
|  | Get cell type of this Cell. More... |
|  | |
| int | SetColour (const int new\_colour) |
|  | Set color of this cell to new\_colour, irrespective of type. More... |
|  | |
| int | EnergyDifference (const Cell &cell2) const |
|  | |
| int | Area () const |
|  | Return Cell's actual area. More... |
|  | |
| int | TargetArea () const |
|  | Return Cell's target area. More... |
|  | |
| double | TargetLength () const |
|  | Return Cell's target length. More... |
|  | |
| double | SetTargetLength (double l) |
|  | Set the Cell's target length. More... |
|  | |
| void | PrintInertia (void) |
|  | Debugging function used to print the cell's current inertia tensor (as used for calculations of the length ) More... |
|  | |
| double | Length (void) |
|  | |
| void | RenormPolarVec (void) |
|  | |
| int | Sigma () const |
|  | Returns the cell's cell identity number. More... |
|  | |
| int | SetTargetArea (const int new\_area) |
|  | Sets the target area of the cell. More... |
|  | |
| void | Apoptose () |
|  | Sends the current cell into apoptosis. More... |
|  | |
| int | IncrementTargetArea () |
|  | Decrement the cell's target area by one unit. More... |
|  | |
| int | DecrementTargetArea () |
|  | Increment the cell's target area by one unit. More... |
|  | |
| int | Mother (void) const |
|  | Cell lineage tracking: get the cell's parent. More... |
|  | |
| int | Daughter (void) const |
|  | Cell lineage tracking: get the cell's daughter. More... |
|  | |
| int | TimesDivided (void) const |
|  | Returns a counter keeping track of the number of divisions. More... |
|  | |
| int | DateOfBirth (void) const |
|  | Returns Monte Carlo Step (MCS) when this cell originated. More... |
|  | |
| int | ColourOfBirth (void) const |
|  | Returns the cell type at the time of birth. More... |
|  | |
| int | GetJ (const Cell &c2) const |
|  | Returns the bond energy J between this cell and cell c2. More... |
|  | |
| double \* | SetGrad (double \*g) |
|  | Set the current gradient of the cell to g. Currently not in use. More... |
|  | |
| const double \* | GetGrad (void) const |
|  | Returns the cell's measured gradient. Currently not in use. More... |
|  | |
| const double | GradX () const |
|  | Returns the cell's measured gradient. Currently not in use. More... |
|  | |
| const double | GradY () const |
|  | Returns the cell's measured gradient. Currently not in use. More... |
|  | |
| double \* | AddToGrad (double \*g) |
|  | Currently not in use (remove?) More... |
|  | |
| void | ClearGrad (void) |
|  | Currently not in use (remove?) More... |
|  | |
| void | MeasureCellSize (Cell &c) |
|  | |

|  |  |
| --- | --- |
| Static Public Member Functions | |
| static void | ClearJ (void) |
|  | Clears the table of J's. More... |
|  | |
| static int | MaxSigma () |
|  | Returns the maximum cell identity number in the Dish. This would normally be the number of cells in the Dish, although the number includes apoptosed cells. More... |
|  | |
| static int | SetJ (int t1, int t2, int val) |
|  | Sets bond energy J between cell type t1 and t2 to val. More... |
|  | |

|  |  |
| --- | --- |
| Public Attributes | |
| double | polarvec [9] |
|  | |

|  |  |
| --- | --- |
| Protected Attributes | |
| int | colour |
|  | |
| bool | alive |
|  | |
| int | sigma |
|  | |
| int | tau |
|  | |
| double | length |
|  | |
| double | target\_length |
|  | |
| int | mother |
|  | |
| int | daughter |
|  | |
| int | times\_divided |
|  | |
| int | date\_of\_birth |
|  | |
| int | colour\_of\_birth |
|  | |
| int | area |
|  | |
| int | target\_area |
|  | |
| int | growth\_threshold |
|  | |
| double | v [2] |
|  | |
| int | n\_copies |
|  | |
| double | grad [2] |
|  | |
| double \* | chem |
|  | |
| long int | sum\_x |
|  | |
| long int | sum\_y |
|  | |
| long int | sum\_xx |
|  | |
| long int | sum\_yy |
|  | |
| long int | sum\_xy |
|  | |
| const Dish \* | owner |
|  | |

|  |  |
| --- | --- |
| Static Protected Attributes | |
| static int \*\* | J =0 |
|  | |
| static int | maxtau =0 |
|  | |
| static int | amount =0 |
|  | |
| static int | capacity =0 |
|  | |
| static int | maxsigma =0 |
|  | |

|  |  |
| --- | --- |
| Friends | |
| class | Dish |
|  | |
| class | CellularPotts |
|  | |
| class | Info |
|  | |

## Constructor & Destructor Documentation

|  |  |  |  |
| --- | --- | --- | --- |
| Cell::Cell | ( | const Dish & | *who*, |
|  |  | int | *settau* = `1` |
|  | ) |  |  |

inline

Constructor to insert a cell into Dish "who".

Used to add a new Cell to the dish: new Cell(dish, celtype).

References owner.

|  |  |  |  |  |  |
| --- | --- | --- | --- | --- | --- |
| Cell::Cell | ( | void |  | ) |  |

inline

References chem, and Parameter::n\_chem.

|  |  |  |  |  |  |
| --- | --- | --- | --- | --- | --- |
| Cell::~Cell | ( | void |  | ) |  |

|  |  |  |  |  |  |
| --- | --- | --- | --- | --- | --- |
| Cell::Cell | ( | const Cell & | *src* | ) |  |

inline

Default copy constructor.

References alive, amount, area, chem, colour\_of\_birth, date\_of\_birth, daughter, growth\_threshold, length, mother, Parameter::n\_chem, n\_copies, owner, sigma, sum\_x, sum\_xx, sum\_xy, sum\_y, sum\_yy, target\_area, target\_length, tau, times\_divided, and v.

## Member Function Documentation

|  |  |  |  |  |  |
| --- | --- | --- | --- | --- | --- |
| double\* Cell::AddToGrad | ( | double \* | *g* | ) |  |

inline

Currently not in use (remove?)

References grad.

|  |  |  |  |  |  |
| --- | --- | --- | --- | --- | --- |
| bool Cell::AliveP | ( | void |  | ) | const |

inline

Returns false if Cell has apoptosed (vanished).

References alive.

|  |  |  |  |  |
| --- | --- | --- | --- | --- |
| void Cell::Apoptose | ( |  | ) |  |

inline

Sends the current cell into apoptosis.

References alive.

|  |  |  |  |  |
| --- | --- | --- | --- | --- |
| int Cell::Area | ( |  | ) | const |

inline

Return Cell's actual area.

References area.

Referenced by CellularPotts::Compactness(), CellularPotts::MeanCellArea(), Dish::MeasureChemConcentrations(), and Info::Menu().

|  |  |  |  |  |  |
| --- | --- | --- | --- | --- | --- |
| void Cell::CellBirth | ( | Cell & | *mother* | ) |  |

Add a new cell to the dish.

Call it as: new Cell(parent, true); mother will be modified for ancestry administration!

Parameters
:   |  |  |
    | --- | --- |
    | settau | Cell type of daughter cell. |

References alive, chem, colour, daughter, grad, Parameter::n\_chem, owner, sigma, target\_length, tau, Dish::Time(), times\_divided, and v.

|  |  |  |  |  |  |
| --- | --- | --- | --- | --- | --- |
| void Cell::ClearGrad | ( | void |  | ) |  |

inline

Currently not in use (remove?)

References grad.

|  |  |  |  |  |  |
| --- | --- | --- | --- | --- | --- |
| void Cell::ClearJ | ( | void |  | ) |  |

static

Clears the table of J's.

This is only important for a feature called "DynamicJ's", where J-values depend on internal states of the cells (such as a genetic network; see e.g. Hogeweg et al. 2000). The current version of TST does not include such functionality.

References EMPTY.

|  |  |  |  |  |  |
| --- | --- | --- | --- | --- | --- |
| int Cell::Colour | ( | void |  | ) | const |

inline

Returns the cell colour.

References tau.

Referenced by Info::Menu().

|  |  |  |  |  |  |
| --- | --- | --- | --- | --- | --- |
| int Cell::ColourOfBirth | ( | void |  | ) | const |

inline

Returns the cell type at the time of birth.

References colour\_of\_birth.

|  |  |  |  |  |  |
| --- | --- | --- | --- | --- | --- |
| int Cell::DateOfBirth | ( | void |  | ) | const |

inline

Returns Monte Carlo Step (MCS) when this cell originated.

References date\_of\_birth.

|  |  |  |  |  |  |
| --- | --- | --- | --- | --- | --- |
| int Cell::Daughter | ( | void |  | ) | const |

inline

Cell lineage tracking: get the cell's daughter.

References daughter.

|  |  |  |  |  |
| --- | --- | --- | --- | --- |
| int Cell::DecrementTargetArea | ( |  | ) |  |

inline

Increment the cell's target area by one unit.

References target\_area.

|  |  |  |  |  |  |
| --- | --- | --- | --- | --- | --- |
| int Cell::EnergyDifference | ( | const Cell & | *cell2* | ) | const |

References sigma, and tau.

|  |  |  |  |  |  |
| --- | --- | --- | --- | --- | --- |
| const double\* Cell::GetGrad | ( | void |  | ) | const |

inline

Returns the cell's measured gradient. Currently not in use.

References grad.

|  |  |  |  |  |  |
| --- | --- | --- | --- | --- | --- |
| int Cell::GetJ | ( | const Cell & | *c2* | ) | const |

inline

Returns the bond energy J between this cell and cell c2.

References J, and sigma.

|  |  |  |  |  |  |
| --- | --- | --- | --- | --- | --- |
| int Cell::getTau | ( | void |  | ) |  |

inline

Get cell type of this Cell.

References tau.

|  |  |  |  |  |
| --- | --- | --- | --- | --- |
| const double Cell::GradX | ( |  | ) | const |

inline

Returns the cell's measured gradient. Currently not in use.

References grad.

|  |  |  |  |  |
| --- | --- | --- | --- | --- |
| const double Cell::GradY | ( |  | ) | const |

inline

Returns the cell's measured gradient. Currently not in use.

References grad.

|  |  |  |  |  |
| --- | --- | --- | --- | --- |
| int Cell::IncrementTargetArea | ( |  | ) |  |

inline

Decrement the cell's target area by one unit.

References target\_area.

Referenced by Dish::CellGrowthAndDivision().

|  |  |  |  |  |  |
| --- | --- | --- | --- | --- | --- |
| double Cell::Length | ( | void |  | ) |  |

inline

References length.

|  |  |  |  |  |
| --- | --- | --- | --- | --- |
| static int Cell::MaxSigma | ( |  | ) |  |

inlinestatic

Returns the maximum cell identity number in the Dish. This would normally be the number of cells in the Dish, although the number includes apoptosed cells.

References maxsigma.

Referenced by CellularPotts::ReadZygotePicture().

|  |  |  |  |  |  |
| --- | --- | --- | --- | --- | --- |
| void Cell::MeasureCellSize | ( | Cell & | *c* | ) |  |

After introducing a new Cell (e.g. with GrowInCell) call this function to set the moments and areas right.

Referenced by Info::Menu().

|  |  |  |  |  |  |
| --- | --- | --- | --- | --- | --- |
| int Cell::Mother | ( | void |  | ) | const |

inline

Cell lineage tracking: get the cell's parent.

References mother.

|  |  |  |  |  |  |
| --- | --- | --- | --- | --- | --- |
| Cell& Cell::operator= | ( | const Cell & | *src* | ) |  |

inline

Assignment operator.

Called if one cell is assigned to another. Remember to change both assignment operator and copy constructor when adding new attributes to Cell.

References alive, amount, area, chem, colour, length, Parameter::n\_chem, n\_copies, owner, sigma, sum\_x, sum\_xx, sum\_xy, sum\_y, sum\_yy, target\_area, target\_length, tau, and v.

|  |  |  |  |  |  |
| --- | --- | --- | --- | --- | --- |
| void Cell::PrintInertia | ( | void |  | ) |  |

inline

Debugging function used to print the cell's current inertia tensor (as used for calculations of the length )

References area, sum\_x, sum\_xx, sum\_xy, sum\_y, and sum\_yy.

|  |  |  |  |  |  |
| --- | --- | --- | --- | --- | --- |
| void Cell::RenormPolarVec | ( | void |  | ) |  |

|  |  |  |  |  |  |
| --- | --- | --- | --- | --- | --- |
| int Cell::SetColour | ( | const int | *new\_colour* | ) |  |

inline

Set color of this cell to new\_colour, irrespective of type.

References colour.

|  |  |  |  |  |  |
| --- | --- | --- | --- | --- | --- |
| double\* Cell::SetGrad | ( | double \* | *g* | ) |  |

inline

Set the current gradient of the cell to g. Currently not in use.

References grad.

|  |  |  |  |
| --- | --- | --- | --- |
| static int Cell::SetJ | ( | int | *t1*, |
|  |  | int | *t2*, |
|  |  | int | *val* |
|  | ) |  |  |

inlinestatic

Sets bond energy J between cell type t1 and t2 to val.

References J.

|  |  |  |  |  |  |
| --- | --- | --- | --- | --- | --- |
| int Cell::SetTargetArea | ( | const int | *new\_area* | ) |  |

inline

Sets the target area of the cell.

References target\_area.

Referenced by CellularPotts::GrowAndDivideCells(), and Info::Menu().

|  |  |  |  |  |  |
| --- | --- | --- | --- | --- | --- |
| double Cell::SetTargetLength | ( | double | *l* | ) |  |

inline

Set the Cell's target length.

References target\_length.

Referenced by Info::Menu(), and CellularPotts::ResetTargetLengths().

|  |  |  |  |  |  |
| --- | --- | --- | --- | --- | --- |
| void Cell::setTau | ( | int | *settau* | ) |  |

inline

Set cell type of this Cell.

References tau.

Referenced by Info::Menu().

|  |  |  |  |  |
| --- | --- | --- | --- | --- |
| int Cell::Sigma | ( |  | ) | const |

inline

Returns the cell's cell identity number.

References sigma.

Referenced by Info::Menu().

|  |  |  |  |  |
| --- | --- | --- | --- | --- |
| int Cell::TargetArea | ( |  | ) | const |

inline

Return Cell's target area.

References target\_area.

Referenced by Info::Menu().

|  |  |  |  |  |
| --- | --- | --- | --- | --- |
| double Cell::TargetLength | ( |  | ) | const |

inline

Return Cell's target length.

Length constraint is documented in Merks et al. 2006, Dev. Biol.

References target\_length.

|  |  |  |  |  |  |
| --- | --- | --- | --- | --- | --- |
| int Cell::TimesDivided | ( | void |  | ) | const |

inline

Returns a counter keeping track of the number of divisions.

References times\_divided.

## Friends And Related Function Documentation

friend class CellularPotts

friend

friend class Dish

friend

friend class Info

friend

## Member Data Documentation

bool Cell::alive

protected

Referenced by AliveP(), Apoptose(), Cell(), CellBirth(), and operator=().

int Cell::amount =0

staticprotected

Referenced by Cell(), and operator=().

int Cell::area

protected

Referenced by Area(), Cell(), operator=(), and PrintInertia().

int Cell::capacity =0

staticprotected

double\* Cell::chem

protected

Referenced by Cell(), CellBirth(), Dish::MeasureChemConcentrations(), and operator=().

int Cell::colour

protected

Referenced by CellBirth(), operator=(), and SetColour().

int Cell::colour\_of\_birth

protected

Referenced by Cell(), and ColourOfBirth().

int Cell::date\_of\_birth

protected

Referenced by Cell(), and DateOfBirth().

int Cell::daughter

protected

Referenced by Cell(), CellBirth(), and Daughter().

double Cell::grad[2]

protected

Referenced by AddToGrad(), CellBirth(), ClearGrad(), GetGrad(), GradX(), GradY(), and SetGrad().

int Cell::growth\_threshold

protected

Referenced by Cell().

int \*\* Cell::J =0

staticprotected

Referenced by GetJ(), and SetJ().

double Cell::length

protected

Referenced by Cell(), Length(), and operator=().

int Cell::maxsigma =0

staticprotected

Referenced by Dish::ConstructorBody(), and MaxSigma().

int Cell::maxtau =0

staticprotected

Referenced by CellularPotts::SetRandomTypes().

int Cell::mother

protected

Referenced by Cell(), and Mother().

int Cell::n\_copies

protected

Referenced by Cell(), and operator=().

const Dish\* Cell::owner

protected

Referenced by Cell(), CellBirth(), operator=(), and Dish::SetCellOwner().

double Cell::polarvec[9]

int Cell::sigma

protected

Referenced by Cell(), CellBirth(), EnergyDifference(), GetJ(), operator=(), and Sigma().

long int Cell::sum\_x

protected

Referenced by Cell(), operator=(), and PrintInertia().

long int Cell::sum\_xx

protected

Referenced by Cell(), operator=(), and PrintInertia().

long int Cell::sum\_xy

protected

Referenced by Cell(), operator=(), and PrintInertia().

long int Cell::sum\_y

protected

Referenced by Cell(), operator=(), and PrintInertia().

long int Cell::sum\_yy

protected

Referenced by Cell(), operator=(), and PrintInertia().

int Cell::target\_area

protected

Referenced by Cell(), DecrementTargetArea(), IncrementTargetArea(), operator=(), SetTargetArea(), and TargetArea().

double Cell::target\_length

protected

Referenced by Cell(), CellBirth(), operator=(), SetTargetLength(), and TargetLength().

int Cell::tau

protected

Referenced by Cell(), CellBirth(), Colour(), EnergyDifference(), getTau(), operator=(), and setTau().

int Cell::times\_divided

protected

Referenced by Cell(), CellBirth(), and TimesDivided().

double Cell::v[2]

protected

Referenced by Cell(), CellBirth(), and operator=().

---

The documentation for this class was generated from the following files:

- cell.h
- cell.cpp


---

Generated on Thu Aug 14 2014 22:04:01 for Tissue Simulation Toolkit by  

 1.8.6
